# De Novo Dissecting the Three-Dimensional Facial Morphology of 2379 Han Chinese Individuals

**DOI:** 10.1007/s43657-023-00109-x

**Published:** 2023-06-08

**Authors:** Hui Qiao, Jingze Tan, Shaoqing Wen, Menghan Zhang, Shuhua Xu, Li Jin

**Affiliations:** 1 State Key Laboratory of Genetic Engineering, Center for Evolutionary Biology, Collaborative Innovation Center for Genetics and Development, School of Life Sciences, Fudan University, Shanghai, 200438 China; 2 Ministry of Education Key Laboratory of Contemporary Anthropology, Human Phenome Institute, Zhangjiang Fudan International Innovation Center, Fudan University, Shanghai, 201203 China; 3 Institute of Archaeological Science, Fudan University, Shanghai, 200433 China; 4 Institute of Modern Languages and Linguistics, Fudan University, Shanghai, 200433 China; 5 Department of Liver Surgery and Transplantation Liver Cancer Institute, Zhongshan Hospital, Fudan University, Shanghai, 200032 China

**Keywords:** Phenotypes, Three-dimensional facial imaging, Facial morphology, Han Chinese

## Abstract

Phenotypic diversity, especially that of facial morphology, has not been fully investigated in the Han Chinese, which is the largest ethnic group in the world. In this study, we systematically analyzed a total of 14,838 facial traits representing 15 categories with both a large-scale three-dimensional (3D) manual landmarking database and computer-aided facial segmented phenotyping in 2379 Han Chinese individuals. Our results illustrate that homogeneous and heterogeneous facial morphological traits exist among Han Chinese populations across the three geographical regions: Zhengzhou, Taizhou, and Nanning. We identified 1560 shared features from extracted phenotypes, which characterized well the basic facial morphology of the Han Chinese. In particular, heterogeneous phenotypes showing population structures corresponded to geographical subpopulations. The greatest facial variation among these geographical populations was the angle of glabella, left subalare, and right cheilion (*p* = 3.4 × 10^−161^). Interestingly, we found that Han Chinese populations could be classified into northern Han, central Han, and southern Han at the phenotypic level, and the facial morphological variation pattern of central Han Chinese was between the typical differentiation of northern and southern Han Chinese. This result was highly consistent with the results revealed by the genetic data. These findings provide new insights into the analysis of multidimensional phenotypes as well as a valuable resource for further facial phenotype-genotype association studies in Han Chinese and East Asian populations.

**Supplementary Information:**

The online version contains supplementary material available at 10.1007/s43657-023-00109-x.

## Introduction

The morphological diversity of the human face is the basis of anthropological studies, and the human face is a highly complex and variable structure resulting from the intricate coordination of numerous factors (Liu et al. 2021). Dissecting and quantifying facial morphological diversity plays an important role in understanding the homogeneity and heterogeneity both within and among various isolated populations or geographical regions. The Han Chinese is the largest ethnic group in the world, and their origins, formation, and development are complex and lengthy. To explain the development history of the Han Chinese over 2000 years, historians, geneticists, and anthropologists have studied the population structure of the Han Chinese according to different perspectives. Previous study proposed that the Han Chinese were relatively genetically homogeneous (Liu et al. 2018). A cluster analysis of dermatoglyphics indicated that Han Chinese samples from different places tended to cluster together as a group with local minorities. The Chinese nation has been diversified and integrated, and the Han Chinese were the offspring of the Chinese nation (Zhang et al. 2010a, b). In contrast, many genetic studies have found that the Han Chinese could be divided into several distinct groups. A study showed that the Han Chinese could be distinguished according to three clusters corresponding roughly to northern Han, central Han, and southern Han with 160,000 single-nucleotide polymorphisms (Xu et al. 2009). The classification results for these three subpopulations also were verified in two independent studies using different datasets (Chen et al. 2009; Liu et al. 2018). In contrast to these conclusions, another recent study explored the Chinese population structure with whole-genome sequencing and Asian screening array and found that the Han Chinese could be classified into four subgroups: northern Han, central Han, southern Han, and Lingnan Han (Cong et al. 2022). Moreover, most scholars believed that the Han Chinese could be divided into the south and the north in terms of their physical characteristics (Liu 1991; Zhang 1988). It has not been proved, however, whether central Han exists at the phenotypic level as genetics have become more understood.

In this study, we analyzed the Han ethnic groups in three geographical regions according to two morphological dissecting approaches. We demonstrated that the Han Chinese were first and foremost a unity. People living in different regions shared some homogenous morphological traits, but at the same time, a transitional subpopulation existed between northern Han and southern Han, called the central Han, whose facial morphological traits were mostly between northern and southern Han, and were closer to the northern Han population on the whole. To our knowledge, this is the first time that such a detailed and comprehensive dissection of facial traits has been conducted in Han Chinese populations by a large three-dimensional (3D) facial images cohort. These findings likely support implications for fundamental and applied sciences, including human genetics, developmental biology, evolutionary biology, medical genetics, forensics, and the design of facial products.

## Materials and Methods

### Samples and Recruitment Details

**Fig. 1:**
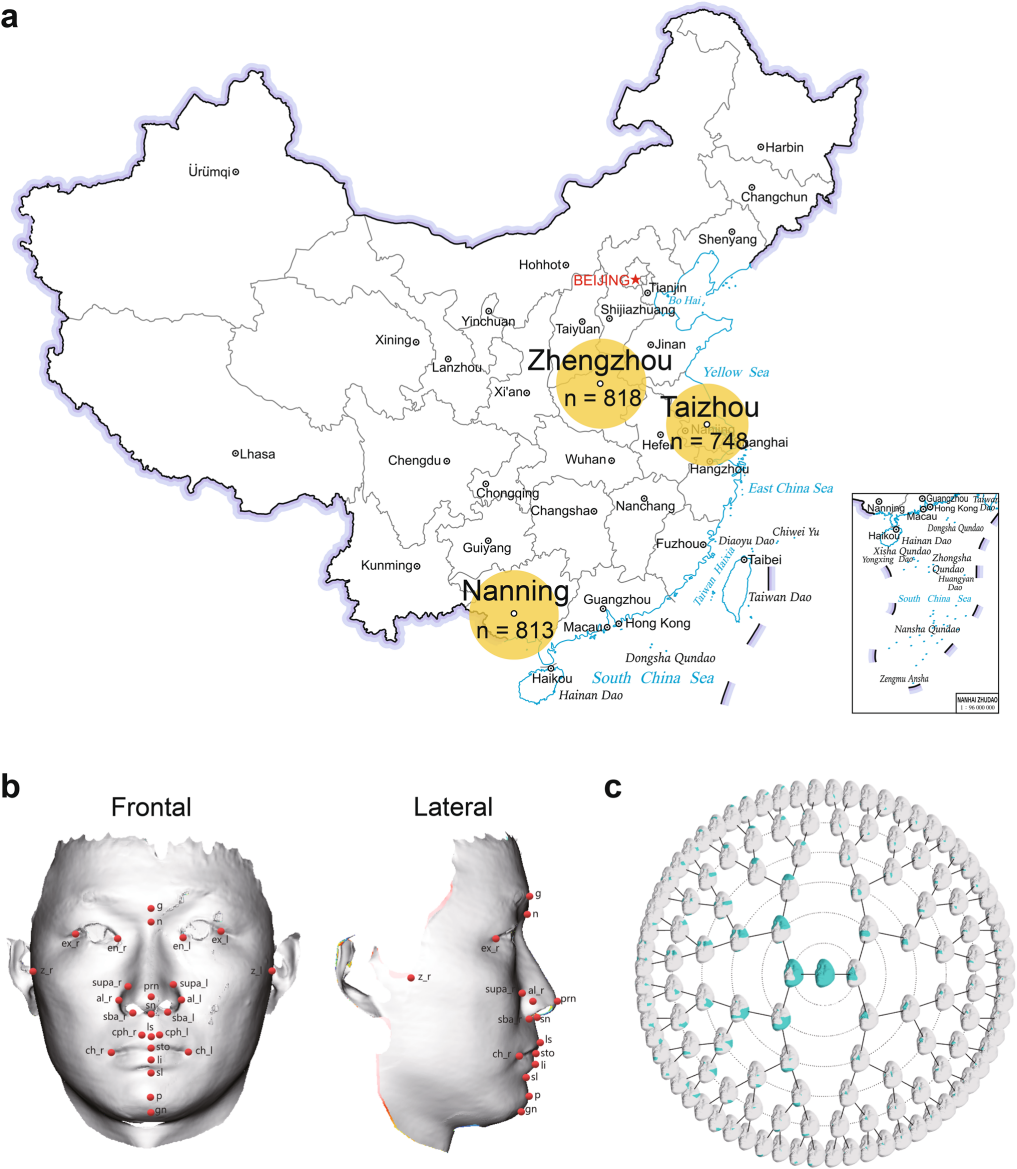
Sample distribution and types of phenotypes. **a** Geographical distribution of sample collection. **b** Landmarks used for morphometric analysis of facial shape in manual landmarking approach.** c** Hierarchical spectral clustering of facial shape in computer-aided approach. The geographical base map was obtained from the National Platform for Common Geospatial Information Services

We completed data collection between 2015 and 2019. The repository included 3D facial surface images and self-reported demographic descriptors as well as basic physical characteristics from participants. Raw 3D facial images of the participants were collected by a GFM FaceScan 3D System (GFMesstechnik GmbH, Teltow, Germany), which captured a 3D surface map in about 0.15 s, with a high-resolution color surface generated with approximately 50,000 vertices. Subjects were seated on an adjustable chair in front of the equipment with the Frankfort plane raised upward by about 10° to the horizontal level to ensure a full image of the nose and jaw. Subjects were instructed to relax with their lips together without straining and with their eyes open without stretching the forehead (Bugaighis et al. 2013). To ensure that the 3D images were unobscured by the hair and to capture the shape of the forehead and ears, we used a hair clasp and natural head postures for all subjects. Participants were recruited from the following three Chinese cities: Taizhou city of Jiangsu Province, Zhengzhou city of Henan Province, and Nanning city of Guangxi Zhuang autonomous region. The three regions are typical of northern, central, and southern Han Chinese in terms of genetic structure. To minimize the effects of ethnic variability on the facial measurements and analysis, we restricted samples to the Han Chinese in the local place who did not have a history of craniofacial trauma, congenital malformation, or surgery. We retained a total of 2379 participants (904 males, 1475 females) for analysis after removing any participants who were missing personal information or with 3D image artifacts. Our study included 2379 participants from the following three regions: Taizhou, Zhengzhou, and Nanning (Fig. 1a). Of these, 748 were from Taizhou, including 264 males and 484 females; 818 were from Zhengzhou, including 314 males and 504 females; and 813 were from Nanning, including 326 males and 487 females. The gender ratio was similar among the three regions. The participants' ages ranged from 17 to 83 years, with a mean age of 48.9 ± 12.7 years. The mean age for males was 49.7 ± 13.0 years, while the mean age for females was 48.3 ± 12.4 years. All participants provided written informed consent for academic use of the data, before participating in the study. Sample collection for this study was conducted with the approval of the ethics committee of School of Life Sciences at Fudan University.

### Preprocessing of the 3D Data

We preprocessed all 3D images with the GFM FaceScan 3D System for image cropping and pose normalization. First, we cropped and trimmed the surfaces to remove nonfacial areas, such as the neck, hair, and clothes. Second, we performed a pose normalization function, and calibrated all faces to a common reference coordinate system. Because the original scan data had some extra points or missing data on the top of the head, nose, and eye holes, these noise points were cleaned, and the holes were filled with a *possion* function in MeshLab before further data processing (Kazhdan and Hoppe 2013). We defined 26 soft tissue landmarks according to Farkas (1994), which were manually landmarked on each 3D facial surface using MATLAB R2016b (Fig. 1b and Supplementary Table 1) (The MathWorks 2016). Each sample was landmarked twice in sessions conducted two weeks apart to reduce the potential for memory bias by a single trained operator. We recorded the *x*, *y*, and *z* coordinates of each landmark. The average of the two manual landmarking coordinates eventually was used for subsequent analysis. We performed generalized Procrustes analysis (GPA) to align the sets of 26 facial landmarks by removing translation and rotation (Bookstein 1992). We did not perform scaling to preserve the different face sizes.

### Dissecting Facial Phenotypes

We used RStudio 3.5.1 (R Team 2013) and MATLAB R2016b to extract various facial measurements. Our analysis of 3D manual landmarking measurements was based on 26 landmarks, including point coordinates, mean curvatures, Gaussian curvatures, Euclidean distances, Manhattan distances, geodesic distances (Surazhsky et al. 2005), proportion indices, angular measurements, triangle area measurements, voluminal measurements, and surface area measurements.

Curvature is a directional property and describes how bent a surface is around each point (Roberts 2001). The curvature magnitude of one point in some direction is given by the reciprocal of the radius of the circle that best approximates the slice of the surface in that direction (Koenderink and van Doorn 1992). We defined normal curvatures on orthogonal planes to a surface point and for each such point. The largest absolute normal curvature magnitude was called the maximum curvature *K*_max_. The curvature perpendicular to *K*_max_ was the minimum curvature of *K*_min_. Mean curvature significantly differentiated areas of high and low curvature, as well as convex and concave shapes. Gaussian curvature discriminated well between spherical and saddle-like areas (Tsagkrasoulis et al. 2017). The mean curvature and Gaussian curvature are calculated as follows:

1 $${\text{MC}} = \frac{{K_{\max } + K_{\min } }}{2},\;{\text{and}}$$

2 $${\text{GC}} = K_{\max } \times K_{\min } .$$

In this study, we considered three different types of distance-based phenotypes. After GPA, a total of 325 Euclidean distances, Manhattan distances, and geodesic distances (Deschamps and Cohen 2001; Sethian 1999) between all pairs of the 26 landmarks were quantitatively derived, respectively, as follows:

3 $$d = \sqrt[2]{{(a - b)^{T} (a - b)}},$$

4 $$d = \mathop \sum \limits_{k = 1}^{n} |a_{k} - b_{k} |,\;{\text{and}}$$

5 $$\left\{ {\begin{array}{*{20}l} {U(p) = \mathop {\inf }\limits_{{A_{{p_{0} ,p}} }} E(C) = \mathop {\inf }\limits_{{A_{{p_{0} ,p}} }} \left\{ {\int\limits_{\Omega } {\tilde{P}(C(s)){\text{d}}s} } \right\}} \hfill \\ {(\max \{ u - U_{i - 1,j,k} ,u - U_{i + 1,j,k} ,0\} )^{2} } \hfill \\ { + (\max \{ u - U_{i,j - 1,k} ,u - U_{i,j + 1,k} ,0\} )^{2} } \hfill \\ { + (\max \{ u - U_{i,j,k - 1} ,u - U_{i,j,k + 1} ,0\} )^{2} = \tilde{P}_{i,j,k}^{2} } \hfill \\ \end{array} } \right..$$

Euclidean distances have been used to provide a battery of individual facial ratios, which are presented as proportion indices (Farkas 1987) and involve two linear measurements (the smaller expressed as a percentage of the larger). Finally, we selected 32 of the proportion indices (Farkas 1987).

Angular measurements refer to the angle made from two lines through three landmarks in Euclidean space (Maths). We calculated 7800 angles through 26 landmarks. We used the following formula and RStudio to calculate the angle as follows:

6 $$\theta = {\text{cos}}^{ - 1} \frac{{\vec{A}\vec{B}}}{{\left| {\vec{A}} \right|\left| {\vec{B}} \right|}}.$$

The area of a triangle was defined as the total region that was enclosed by the three sides of any particular triangle. We extracted 2600 triangle areas by 26 landmarks. The area of a triangle with three sides of different measures can be found using Heron's formula (Maths) as follows:

7 $$A = \sqrt {s(s - a)(s - b)(s - c)} ,$$

where *s* is the semiperimeter of the triangle, *s* = (*a* + *b* + *c*)/2, and *a*, *b*, *c* are the three sides of a triangle.

We extracted the volume and surface area of a polyhedron, as implemented in the function *surfaceArea* and *convHull* from MATLAB 2016b, respectively, as 3D phenotypes. The polyhedron consisted of several landmarks. We calculated 10 volumes or surface areas, including cheek, nose, chin, and mouth.

We processed and segmented traits derived according to the computer-aided approach from a previous study (Claes et al. 2018). As a result, the global-to-local facial phenotyping partitioned the facial shapes into 127 facial segments, each of which consisted of several point clouds and was represented by multiple dimensions of variation (Fig. 1c). The extracted phenotypes included the principal components of the module, surface area of the module, Moran's *I* of module *Z* coordinate, Moran's *I* of module mean curvature, and Moran's *I* of module Gaussian curvature.

We applied principal component analysis (PCA) to extract the major factors of shape variation characterizing each facial segment. We used parallel analysis (Hayton et al. 2004) to determine the number of principal components needed to adequately summarize the shape variations for a given segment (Claes et al. 2018). In this way, we extracted a total of 2773 principal component phenotypes.

We calculated each segment, as implemented in the function *meshSurfaceArea* from MATLAB 2016b, with its 3D surface area. We included 127 surface areas of modules in the final facial phenotype library.

Moran's *I* is one of the most common indicators of global clustering. Developed by Patrick Alfred Pierce Moran, it examines whether nearby areas have similar or dissimilar attributes overall (1950). If just one variable or attribute is under consideration, the formula is as follows:

8 $$I=\frac{n}{\sum_{i=1}^{n}\sum_{j=1}^{n}{w}_{ij}}\times \frac{\sum_{i=1}^{n}\sum_{j=1}^{n}{w}_{ij}({x}_{i}-\overline{x })({x}_{j}-\overline{x })}{\sum_{i=1}^{n}{({x}_{i}-\overline{x })}^{2}},$$

where *n* is the total number of observations (points or polygons); *i* and *j* represent different locations, *x*_*i*_ and *x*_*j*_ are values of the variable in the *i*th and *j*th locations; and $\overline{x }$ is the mean of the variable, and *w*_*ij*_ is a measure of spatial proximity for pairs *i* and *j* (Xu and Kennedy 2015). We chose *z* coordinate, mean curvature, and Gaussian curvature as the attribute value variables. The *x* and *y* coordinates represented each landmark's different locations. We used the *spdep* package (Bivand 2022) in R for the Moran's *I* procedures. Finally, we obtained 127 Moran's *I* of module *Z* coordinate, Moran's *I* of module mean curvature, and Moran's *I* of module Gaussian curvature.

### Quality Control

Quality control started with 3D image acquisition and questionnaires. The staff at each recruitment site were trained on proper acquisition techniques as well as how to evaluate 3D facial surfaces for quality and coverage (Heike et al. 2010). Each image was generated within 1–2 min and was reviewed immediately to ensure the absence of acquisition errors, such as imaging artifacts, blurring, absence of surface data, or the lack of a neutral facial expression. We deleted images with the incorrect characteristics, and took new images to ensure that they fit the set criteria (Aynechi et al. 2011). To increase the accuracy and reproducibility of facial landmarking, landmarks that required palpation for identification were directly labeled by an experienced operator using an erasable marking pen before image acquisition (Aynechi et al. 2011).

### Statistical Analysis

All tests of hypotheses were two-sided (*α* = 0.05). We compared the average difference of *x*, *y*, and *z* coordinates between twice manual landmarking to evaluate the repeatability. The reliability coefficient of facial traits between spot sampling and 3D manual landmarking further proved the credibility of manual landmarking. We calculated intraclass correlation coefficient (ICC) estimates and their 95% confident intervals using R package *irr* (Gamer et al. 2019) based on multiple raters, consistency, and a two-way mixed-effects model. We used χ^2^ test to evaluate the gender ratio among different regions. We used the Spearman rho correlation, a nonparametric correlate of Pearson correlation, to analyze the facial parameters within the regional difference. Corrections were made for multiplicity using the Bonferroni method to reduce the likelihood of Type 1 errors; for example, an α threshold for statistical significance for 14,838 comparisons was determined to be 3.37 × 10^−6^ (i.e., *α* = 0.05/14,838). To investigate the difference between subgroups and discriminative variable selection, we performed a partial least square discriminant analysis (PLS-DA) (Lee et al. 2018) using the SMICA-P software, version 14.0 (Umetrics, Umea, Sweden). To identify the variables responsible for the separation of the groups, we used variable importance in projection (VIP) values. We used VIP score for variable selection because they give the discriminatory power of each variable (Wheelock and Wheelock 2013). VIP values > 1.0 indicated the maximum discriminatory power, whereas those with values < 1.0 indicated minimal discriminatory power (Chong and Jun 2005). We further used permutational multivariate analysis of variance (PERMANOVA, also known as Adonis analysis) to analyze the explanatory degree of different grouping factors for the sample differences, and we conducted statistical tests on the grouping factors (Anderson 2001). We performed Adonis using the *Vegan* package (Oksanen et al. 2022) in R.

## Results

### Reproducibility of Landmark Identification

As would be expected, one person's perception in terms of landmark reproducibility was better than that of other assessors (Gwilliam et al. 2006). In this study, we obtained 3D manual landmark data by one operator placing the 26 landmarks on all 2379 facial images twice. The overall reproducibility of each landmark is shown in Supplementary Fig. 1 and Supplementary Table 2 in which 55 out of the 78 *x*, *y*, and *z* coordinates had moderate or high reproducibility (SD ≤ 1 mm). The results showed that some landmarks were less reproducible than others. The best reproducibility was the *z* coordinate of pronasale (SD = 0.16), and the worst was the *z* coordinate of the left zygion (SD = 4.09). Notably, the poorer reproducibility of zygion on the lateral view was in agreement with two previous studies (Gwilliam et al. 2006; Hajeer et al. 2002), which was a result of the difficulty in locating these points precisely on the screen. In addition, some facial landmarks showed a greater degree of reproducibility in different planes of space (*x*, *y*, or *z* coordinates). To further confirm the reliability of 3D facial phenotyping, we selected 19 phenotypes identical to those collected in the sampling sites and conducted a consistency analysis on them. On the basis of the ICC results, the reliability of 3D facial phenotyping was regarded as moderate to excellent in which 13 out of the 19 phenotypes indicated good or excellent reliability (Supplementary Table 3).

### Correlation Analysis Between Facial Phenotypes with Geographical Regions

**Fig. 2:**
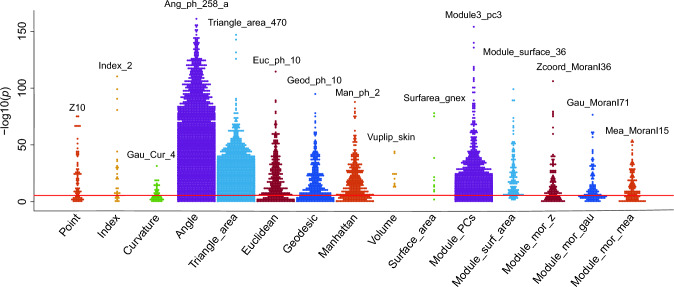
Relationship between facial phenotypes and geographical regions. Each dot represents one phenotype, and the same type of phenotype is indicated in a common color. The solid red line represents multiple correction significance thresholds (*p* = 3.37 × 10^−6^), and phenotypes above the red line denote significant difference or correlation. In each type, the most significant features were the phenotypes of literal labeling. The types of phenotypes are abbreviated as point coordinates (Point), proportion indices (Index), curvatures (Curvature), angular measurements (Angle), triangle area measurements (Triangle_area), Euclidean distances (Euclidean), geodesic distances (Geodesic), Manhattan distances (Manhattan), voluminal measurements (Volume), surface area measurements (Surface_area), principal components of module (Module_PCs), surface area of module (Module_surf_area), Moran's *I* of module *Z* coordinate (Module_mor_z), Moran's *I* of module Gaussian curvature (Module_mor_gau), and Moran's *I* of module mean curvature (Module_mor_mea)

To probe the role of geographical regions in different phenotypes, we analyzed the correlation between regions and facial phenotypes. As shown in Fig. 2, most phenotypes were significantly different among the different regions after multiple adjustments (*p* < 3.37 × 10^−6^). Interestingly, all extracted volume phenotypes were correlated with the region. We observed the biggest correlations with regions for the angle of glabella, left subalare, and right cheilion (*p* = 3.4 × 10^−161^).

### Discriminant Analysis and Facial Features Selection Among Regional Groups

**Fig. 3:**
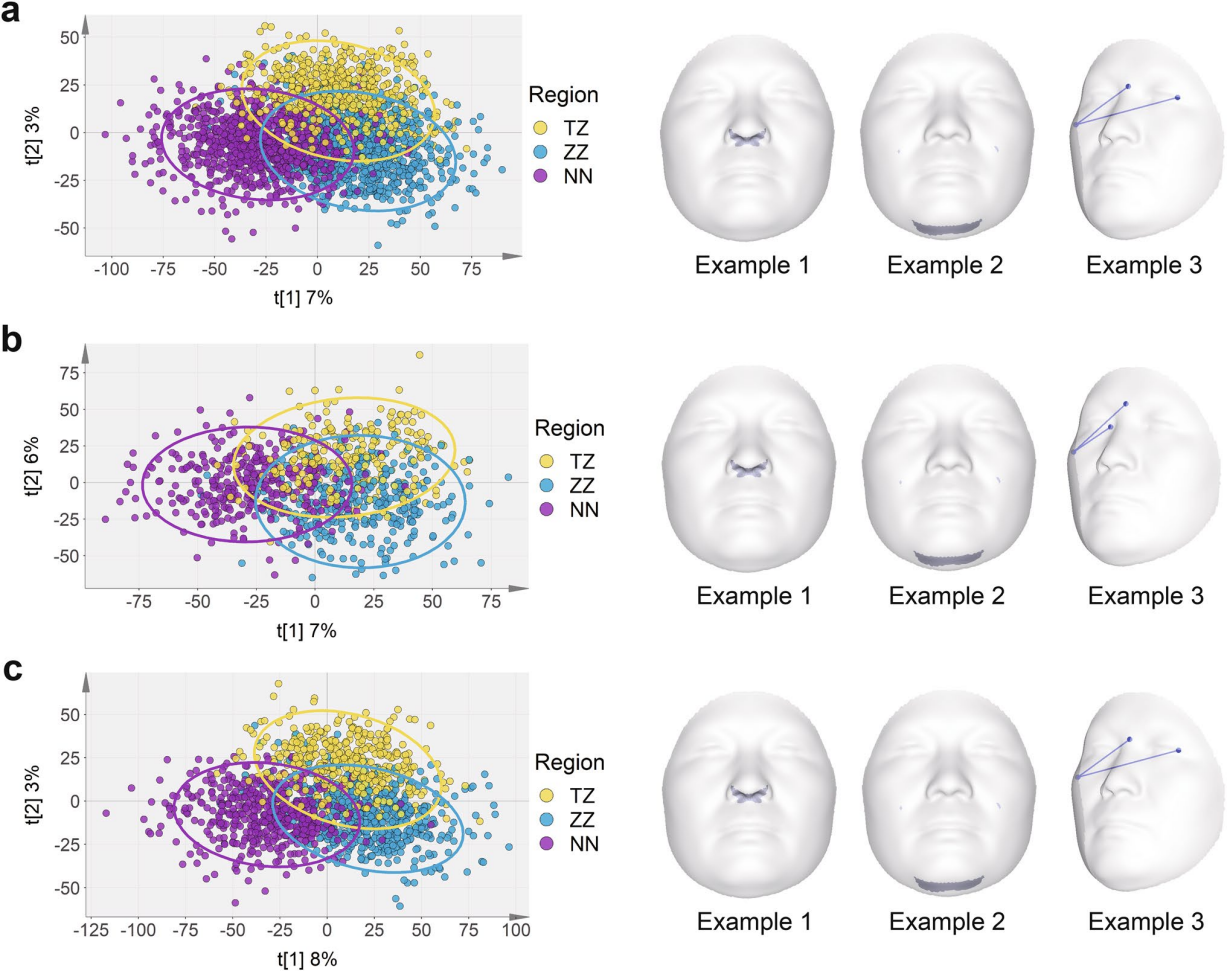
Scatter plot of regional groups and representative facial features. **a** PLS-DA scatter plot model showing separation among regional clustering with individual phenotypic data. Visualization of the variable important in projection (VIP) phenotypes. Example 1: The fourth principal component of the 52nd module. Example 2: The second principal component of the 46th module. Example 3: The angle of nasion, left exocanthion, and right zygion. **b** PLS-DA scatter plot model showing separation among regional clustering with individual phenotypic data in male Han Chinese. Visualization of the VIP phenotypes. Example 1: The fourth principal component of the 52nd module. Example 2: The second principal component of the 46th module. Example 3: The angle of glabella, right endocanthion, and right zygion. **c** PLS-DA scatter plot model showing separation among regional clustering with individual phenotypic data in female Han Chinese. Visualization of the VIP phenotypes. Example 1: The fourth principal component of the 52nd module. Example 2: The second principal component of the 46th module. Example 3: The angle of nasion, left exocanthion, and right zygion. Colored circles represent 95% confidence intervals. Colored dots represent individual samples: Taizhou (yellow), Zhengzhou (blue), and Nanning (purple)

Our study effectively controlled for the gender ratio among different regions during the sample collection process. The gender ratio was found to be similar among the three regions, and χ^2^ test indicated no significant difference in gender ratios among the regions (*p* = 0.14) (Supplementary Fig. 2). To uncover the differences and characteristics of facial phenotypes among regional groups, we relied on PLS-DA as a useful feature selector and classifier. From the scatter plot, three regional groups showed a degree of overlap (Fig. 3a). Nevertheless, PLS-DA (R2X = 0.551, R2Y = 0.728, Q2 = 0.625) and Adonis were able to confirm that Taizhou, Zhengzhou, and Nanning could be separated into different groups (Total: *R*^2^ = 0.05, *p* = 0.001) (Fig. 3a and Supplementary Table 4). The statistical separation among groups confirmed the existence of discriminative features, such as the nose module, chin module, and the angle of nasion, left exocanthion, and right zygion (Fig. 3a). Like the combined data, the male or female samples showed clear separation among the three regional groups, with a slight overlap among the Taizhou, Zhengzhou, and Nanning (Fig. 3b, c). The Adonis analysis also indicated a significant divergence between groups (Supplementary Tables 5 and 6). These results suggested that regional differences existed among the facial phenotypes, and the geographical regions could explain 5% facial variance. To account for the potential impact of age on facial phenotypes, we adjusted for age as a covariate and examined regional differences. Despite controlling for age, the scatter plot still displayed discernible separation among the three regions, and the Adonis analysis confirmed a significant difference among the regions (Supplementary Fig. 3 and Tables 7).

### Heterogeneity of Facial Morphology in Han Chinese

**Fig. 4:**
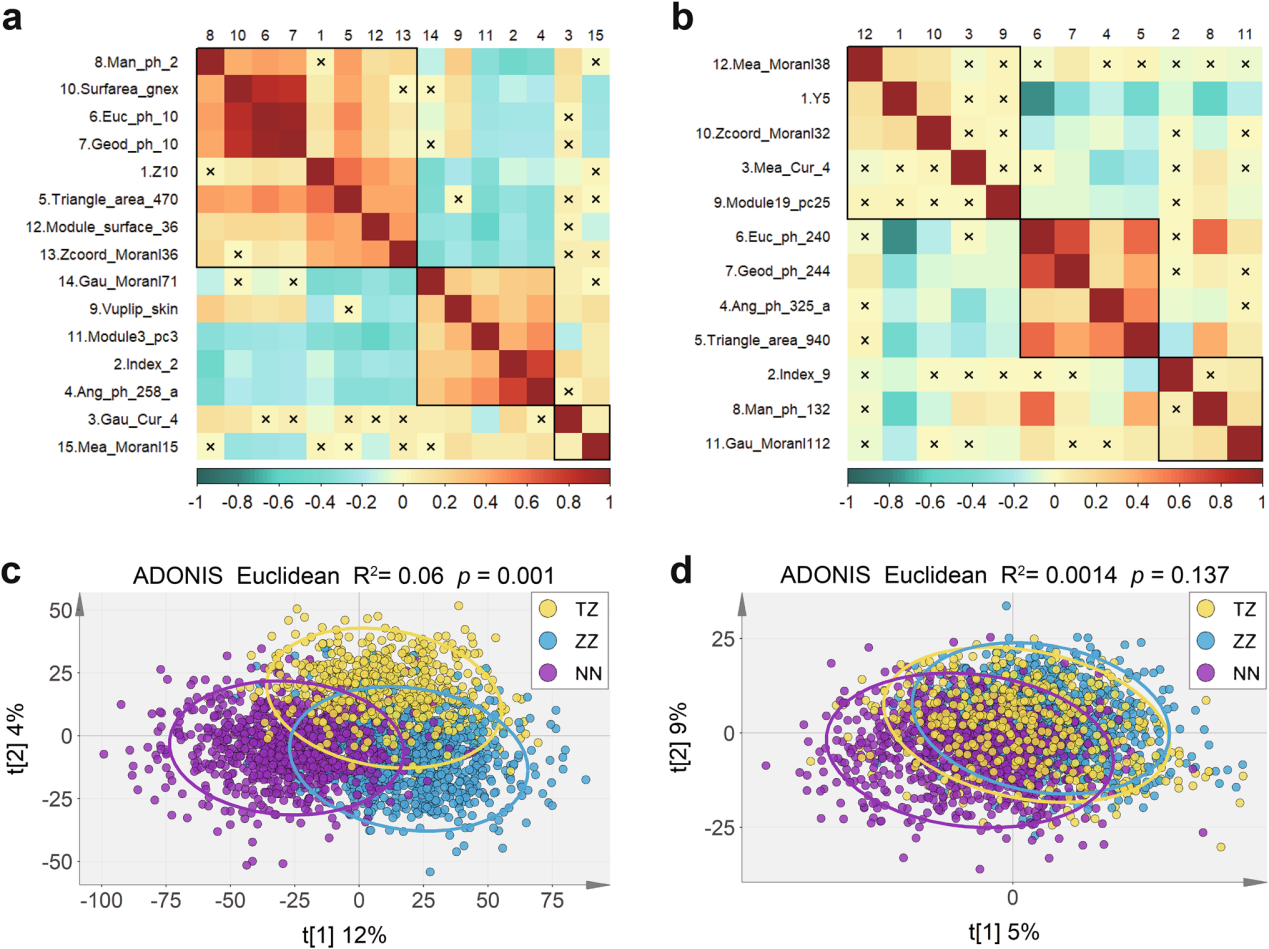
Homogeneity and heterogeneity of facial morphology in Han Chinese. **a** A heat map of the correlation of the heterogeneous features with the smallest *p* value in each type. **b** A heat map of the correlation of the homogeneous features with the smallest *p* value in each type. **c** PLS-DA scatter plot model showing separation trend among regional clustering with heterogeneous facial phenotypes. **d** PLS-DA scatter plot model showing aggregation trend among regional clustering with 1560 homogeneous phenotypes

To examine the potential homogeneous and heterogeneous facial morphological phenotypes in Han Chinese populations, we first used two phenotyping approaches to establish an extensive quantitative and local to global database. By excavating this database, we uncovered a few significant facial phenotypes which were first identified among Han Chinese populations (Fig. 2). We further selected 15 heterogeneous facial phenotypes with the smallest *p* value of each subclass and found that there was a broad association among 15 phenotypes. The greatest correlation was observed between geodesic distance of glabella and left endocanthion (Geod_ph_10) and Euclidean distance of glabella and left endocanthion (Euc_ph_10) (*r* = 0.97) (Fig. 4a). Furthermore, we compared all 15 phenotypes using variance analysis among the three regional groups. We noticed that some phenotypes such as the *z* coordinate of the right endocanthion (Z10), Triangle_area_470, and Euc_ph_10 showed a north–south gradient change (Supplementary Fig. 4). In particular, three regional groups showed distinct morphological characteristics. For example, the Gaussian curvature of subnasale (Gau_Cur_4) was significantly different between Taizhou and Nanning individuals as well as between Taizhou and Zhengzhou individuals, but had no difference between Nanning and Zhengzhou individuals, noting that Gau_Cur_4 in the Taizhou was significantly greater than that in Nanning and Zhengzhou. Indeed, we found that three regional clustering displayed a separation trend with 15 facial morphological phenotypes (Fig. 4c). These results confirmed that facial phenotypes demonstrated heterogeneity in Han Chinese populations. Beyond diversity and heterogeneity, we also investigated homogeneous phenotypes in Han Chinese populations. We examined 1560 homogenous phenotypes among the three regional groups, and PLS-DA scatter plot showed an aggregation trend among regional groups (Fig. 4d). We further analyzed the correlation of 12 homogenous phenotypes with the smallest *p* value in each subtype. These results also showed that a broad correlation existed among these groups (Fig. 4b). In contrast, a violin plot of 12 homogeneous phenotypes revealed no north–south gradient among the three regional groups (Supplementary Fig. 5). Thus, along with previous studies in Han Chinese populations, our data supported the finding that Han Chinese populations had a variety of heterogeneous and homogenous phenotypes, and we confirmed that a central Han population differed from the typical southern Han and northern Han at the facial phenotypic level.

## Discussion

In this study, we identified 15 categories with 14,838 phenotypes from one dimension to three dimensions and used two approaches to dissect facial morphology in Han Chinese populations. Our findings proved that the facial morphology of Han Chinese populations could be categorized into three subgroups: northern Han, central Han, and southern Han.

Population stratification occurs in the presence of undetected population structure whereby study samples composed of sets of individuals differ systematically in both genetic ancestry and the phenotype under investigation (Dempster et al. 2020). Currently, the established knowledge and guidelines related to genetics have been well accepted and applied worldwide (Chen et al. 2009; Cong et al. 2022; Liu et al. 2018; Xu et al. 2009). Interpretation and analysis of phenotypic structures among Han Chinese populations, however, have been lacking. Thus, our dataset is a unique resource and reference for investigating the kinship of populations and the identification of novel phenotype-genotype associations.

According to historical records, the Han Chinese descended from the ancient Huaxia tribes of northern China. In its formation and development, there were three waves of north-to-south migrations during the Western Jin Dynasty (AD 256–316), Tang Dynasty (AD 618–907), and Southern Song Dynasty (AD 1127–1279), respectively (Wen et al. 2004). Our study confirmed that 1560 facial homogeneous phenotypes existed among the three typical regions in the Han Chinese, and heterogeneous phenotypes also existed among different regional groups. A previous study proposed that many southern Han Chinese traced their ancestry to their roots, and often found that the northern Han Chinese who migrated to the south still retained the physical characteristics of the northern Han Chinese after thousands of years (Zheng et al. 2013). Conversely, the northern Han Chinese who migrated to the south communicated and integrated with the ethnic minorities who originally lived in the south, and gradually formed the southern Han Chinese. Genetic studies showed that the greatest differentiation was between the northern and southern samples, and the smallest differentiation was between the northern and central samples (Chen et al. 2009). This result was consistent with our findings at the phenotypic level that the facial morphological traits of the central Han (Taizhou) were closer to the northern Han (Zhengzhou) than to the southern Han (Nanning). These results explained the homogeneous phenotypes among subpopulations in different geographical regions, which can be used to determine the basic characteristics of the Han Chinese. Although the Han Chinese have been formed and developed for thousands of years, heterogeneous phenotypes among the different regions also revealed the diversity within the Han Chinese, which diversified and integrated Han Chinese populations.

The one-dimensional structure of Han Chinese populations was characterized by a continuous genetic gradient along a north–south geographical axis, rather than a distinct clustering of northern and southern samples (Chen et al. 2009). Furthermore, a recent study identified six loci showing genome-wide significance across latitude as follows: *leukocyte immunoglobulin like receptor A3* (*LILRA3*), *complement component 3b/4b receptor 1* (*CR1*), f*atty acid desaturase 2* (*FADS2*), *dedicator of cytokinesis 9* (*DOCK9*), *ATP binding cassette subfamily C member 11* (*ABCC11*), and a cluster of *Immunoglobulin heavy locus* (*IGH*) genes. The *CR1*, *DOCK9*, and the *IGH* genes displayed a higher allele frequency in the south while the *FADS2*, *ABCC11*, and *LILRA3* genes displayed a higher derived allele frequency in the north (Liu et al. 2018). In our study, we also found a south-to-north or north-to-south gradient in facial morphological traits (Supplementary Fig. 4). Combined with the gene frequency gradient, it is reasonable to suspect that the genetic mechanism of facial morphological traits may differ from region to region, and perhaps some phenotypic gradient changes may be related to the gene frequency gradient. Population stratification refers to systematic differences in allele frequencies between subpopulations and is a source of false-positive results in genome-wide association studies (GWAS) (Devlin and Kathryn 1999; Kang et al. 2010; Price et al. 2006; Zhang et al. 2010a, b). Although the proportion of phenotypic variance responsible for the north–south stratification of the Han Chinese was small (just 7% of the total phenotypic variance of the Han Chinese is explained by t1), we still cannot ignore this phenomenon. Just as the first several genetic principal components could be adjusted as covariables when GWAS analysis is involved, when we analyze the phenotypic data of the Han Chinese, we should pay attention to the homogeneity and heterogeneity of facial morphological traits among different geographical regions. In particular, we should reveal the phenotype–genotype associations in the field of medical genetics, consider the population genetic structure, and also recognize the possible population phenotypic structure. It is also important to aware that there are a number of applications of anthropology to the forensic sciences. Human genetic variation is a major resource in forensics, which provides high discriminatory power in identifying known persons, such as perpetrators of crime (Jobling and Gill 2004; Kayser and Knijff 2011). Similarly, the facial morphological traits due to its diversity also have potential applications in forensic anthropology (Mane et al. 2010; Ritz-Timme et al. 2011). Our research showed that the Han Chinese could be divided into northern, central, and southern subgroups, so it is possible to construct mathematical models to speculate one's geographical region. Furthermore, the facial morphological traits may also be used to predict age and gender in the forensic science.

In addition to the variation in facial morphological traits, regional differences were also observed in some disease phenotypes. Previous studies have shown a higher prevalence of hypertension in northern China compared to southern China, mainly attributed to greater body mass index (BMI), higher dietary salt intake, and other lifestyle factors in the northern population (Reynolds et al. 2003). Besides regional differences, a higher number of men were found to be hypertensive than women (29.2% vs 24.1%, *p* < 0.001). The prevalence of hypertension was also observed to increase with age, with young people aged 20–44 years having a prevalence of 13.0%, middle-aged people aged 45–64 years having a prevalence of 36.7%, and elderly people aged 65 years and above having a prevalence of 56.5% (Gao et al. 2013). A study on diabetes in Chinese populations revealed that individuals with type 2 diabetes mellitus (T2DM) in northeast and north China had significantly higher BMI, blood pressure (BP), and low-density lipoprotein cholesterol (LDL-C) levels, and consequently, had high rates of obesity and lower rates of achieving BP and LDL-C targets (Lyu et al. 2018). Furthermore, a national survey of the general population in 2013 (*n* > 0.48 million) showed that northeast Chinese had the highest incidence and mortality rates of stroke, while southwest Chinese had the lowest rates of stroke incidence and mortality (Wang et al. 2017). Understanding the underlying reasons for these variations in disease risk based on population stratifications is crucial in developing targeted prevention and treatment strategies. For example, the northeast and north people should make great efforts to achieve the treatment targets of glycated hemoglobin (HbA1c), BP, and lipid control in treatment of T2DM (Lyu et al. 2018). This approach aligns with the concept of traditional Chinese medicine treatment based on syndrome differentiation and is consistent with the principles of precision medicine.

Although important discoveries have been revealed by these studies, there are also limitations. First, more landmarks need to be included. The 26 landmarks involved in our manual landmarking accounted for only a part of the facial features. For example, the eyes were involved in only two landmarks, and the ears were not involved. Second, our subjects included only three Han Chinese populations, and further research is needed to determine whether these conclusions are generally applicable to other groups.

## Conclusion

In summary, we built the 3D manual landmarking facial morphology database and characterized homogeneous as well as heterogeneous morphological traits in Han Chinese populations. We also provided comprehensive phenotypic evidence showing the stratification of Han Chinese subpopulations, which could be helpful in the study design of an association analysis in Han Chinese populations. Furthermore, the study of population stratification may help embody the traditional Chinese medicine principle of treating the same disease with different methods and treating the different diseases with the same method, just as we use the concept of precision medicine as the basis for personalized therapy.

### Supplementary Information

Below is the link to the electronic supplementary material.

Supplementary file1 (DOCX 10995 KB)

## Data Availability

The datasets generated during and/or analyzed during the current study are available from the corresponding author on reasonable request.

## Abbreviations

3D: Three-dimensional
*ABCC11*: *ATP binding cassette subfamily C member 11*
BMI: Body mass index
BP: Blood pressure
*CR1*: *Complement component 3b/4b receptor 1*
*DOCK9*: *Dedicator of cytokinesis 9*
*FADS2*: *Fatty acid desaturase 2*
GPA: Generalized Procrustes analysis
GWAS: Genome-wide association studies
HbA1c: Glycated hemoglobin
ICC: Intraclass correlation coefficient
*IGH*: *Immunoglobulin heavy locus*
LDL-C: Low-density lipoprotein cholesterol
*LILRA3*: *Leukocyte immunoglobulin like receptor A3*
PCA: Principal component analysis
PERMANOVA: Permutational multivariate analysis of variance
PLS-DA: Partial least square discriminant analysis
T2DM: Type 2 diabetes mellitus
VIP: Variable importance in projection
