# Supplementary material for: De Novo Dissecting the Three-Dimensional Facial Morphology of 2379 Han Chinese Individuals
Source: Phenomics. 2023 Jun 8;4(1):1–12. doi: 10.1007/s43657-023-00109-x (PMC11003940; doi:10.1007/s43657-023-00109-x)
Supplement: Supplementary file 1 — Supplementary file1 (DOCX 10995 KB) [file 43657_2023_109_MOESM1_ESM.docx]

***Supplementary Figures***

**Intra-observer manual landmarking agreement in 3D stereophotogrammetry**


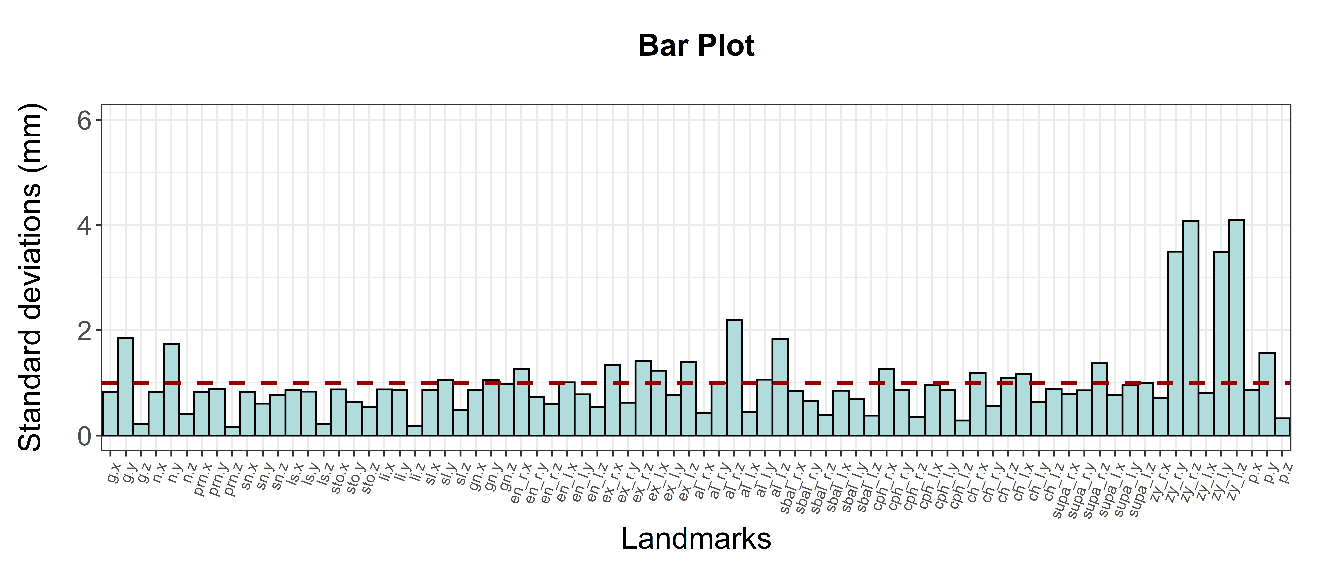


**Supplementary Fig. 1** x, y, and z represent the directions of each landmark on the three coordinate axes. The red dotted line represents SD = 1 mm

**Comparison of gender ratio in each collection site**


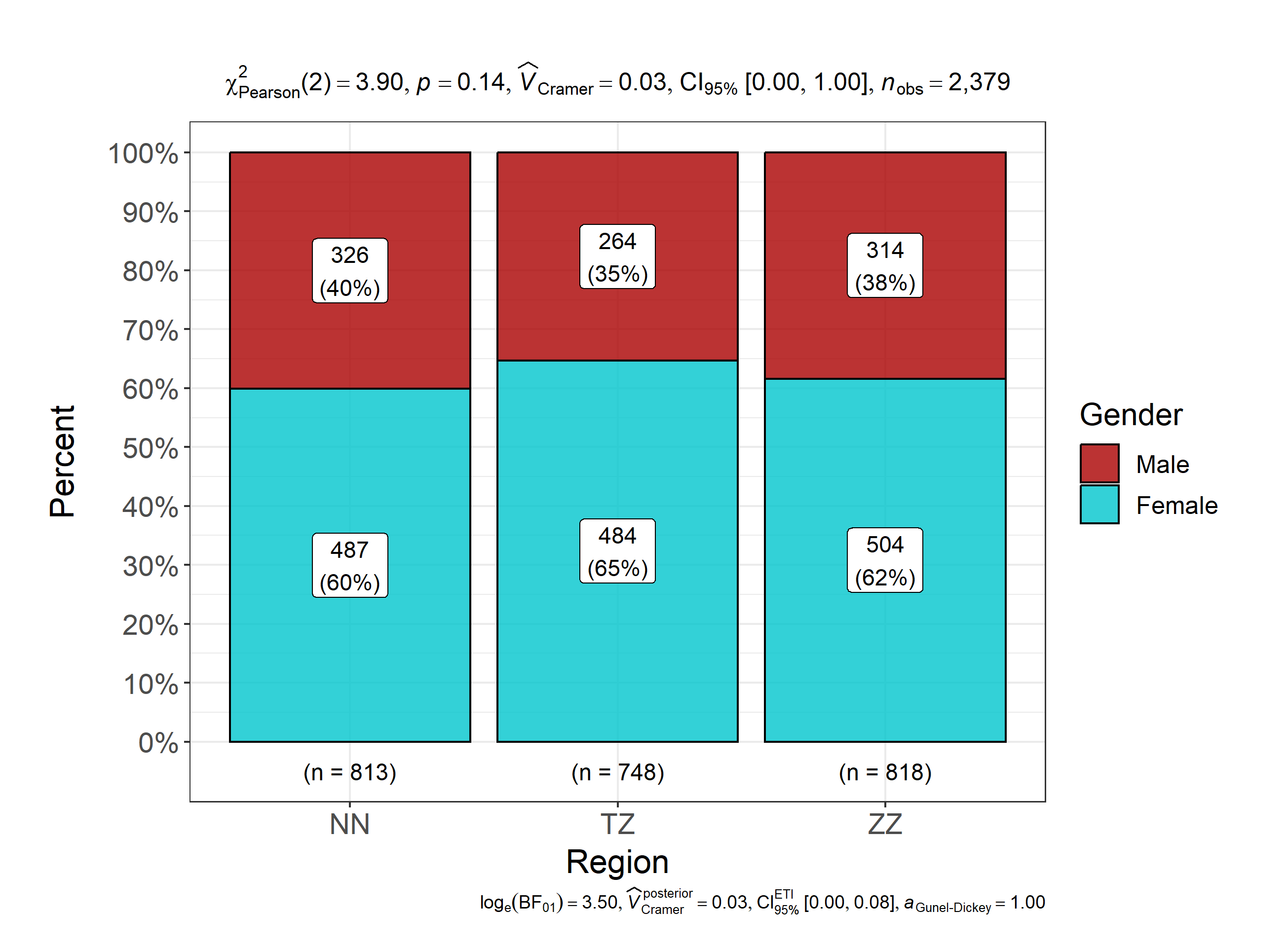

**Supplementary Fig. 2** Bar plot of gender ratio in three regions. Nanning (NN), Taizhou (TZ), and Zhengzhou (ZZ)

**Visualization of the PLS-DA model for regional group after adjusting for age**


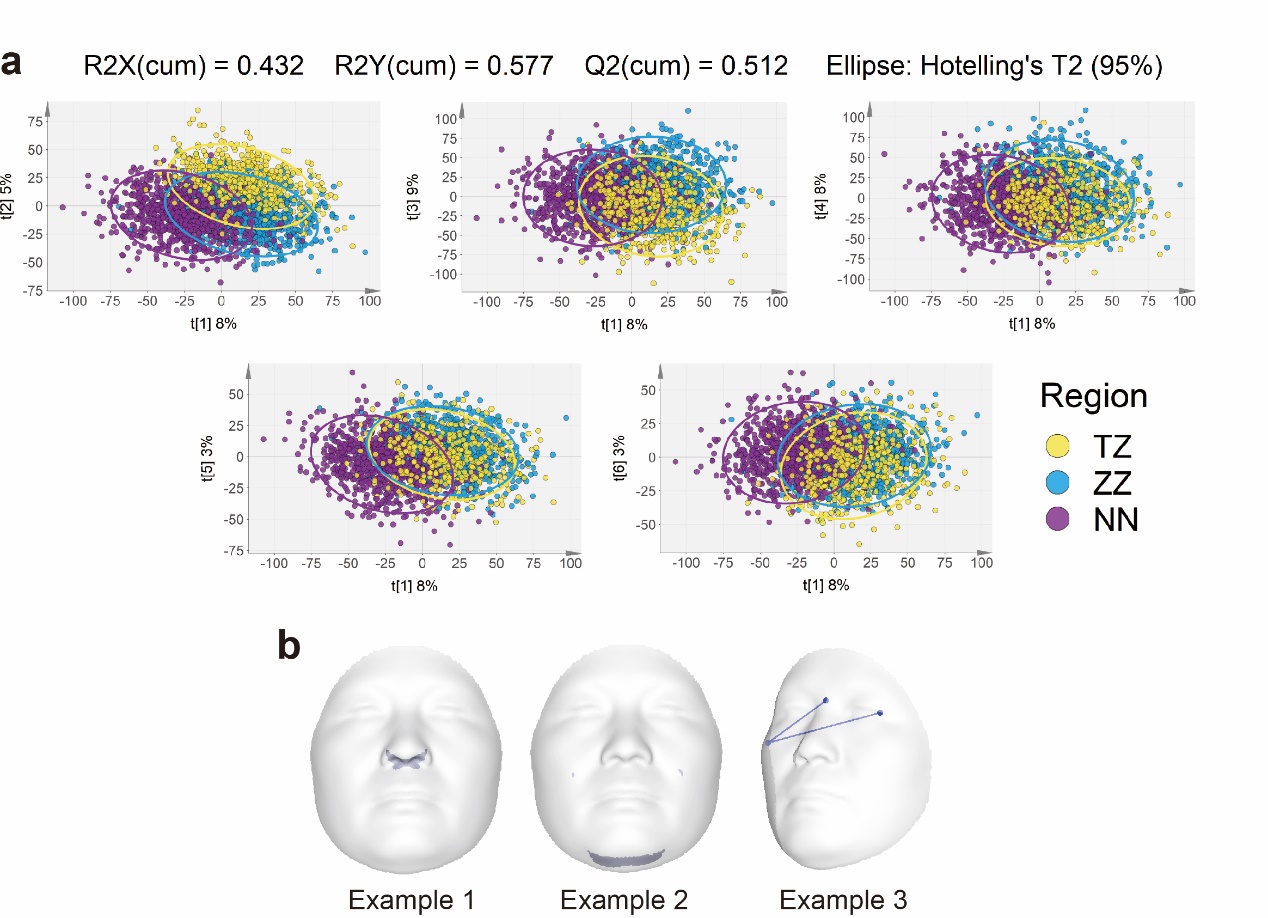


**Supplementary Fig. 3** Scatter plot of regional groups and representative facial features after adjusting for age. **a** PLS-DA scatter plot model showing separation among regional clustering with individual phenotypic data after adjusting for age. **b** Visualization example of the VIP phenotypes. Example 1: The fourth principal component of the 52nd module. Example 2: The second principal component of the 46th module. Example 3: The angle of nasion, left exocanthion, and right zygion. Colored circles represent 95% confidence intervals. Colored dots represent individual samples: Taizhou (yellow), Zhengzhou (blue) and Nanning (purple)

**Heterogeneity of facial features among Han Chinese**

**
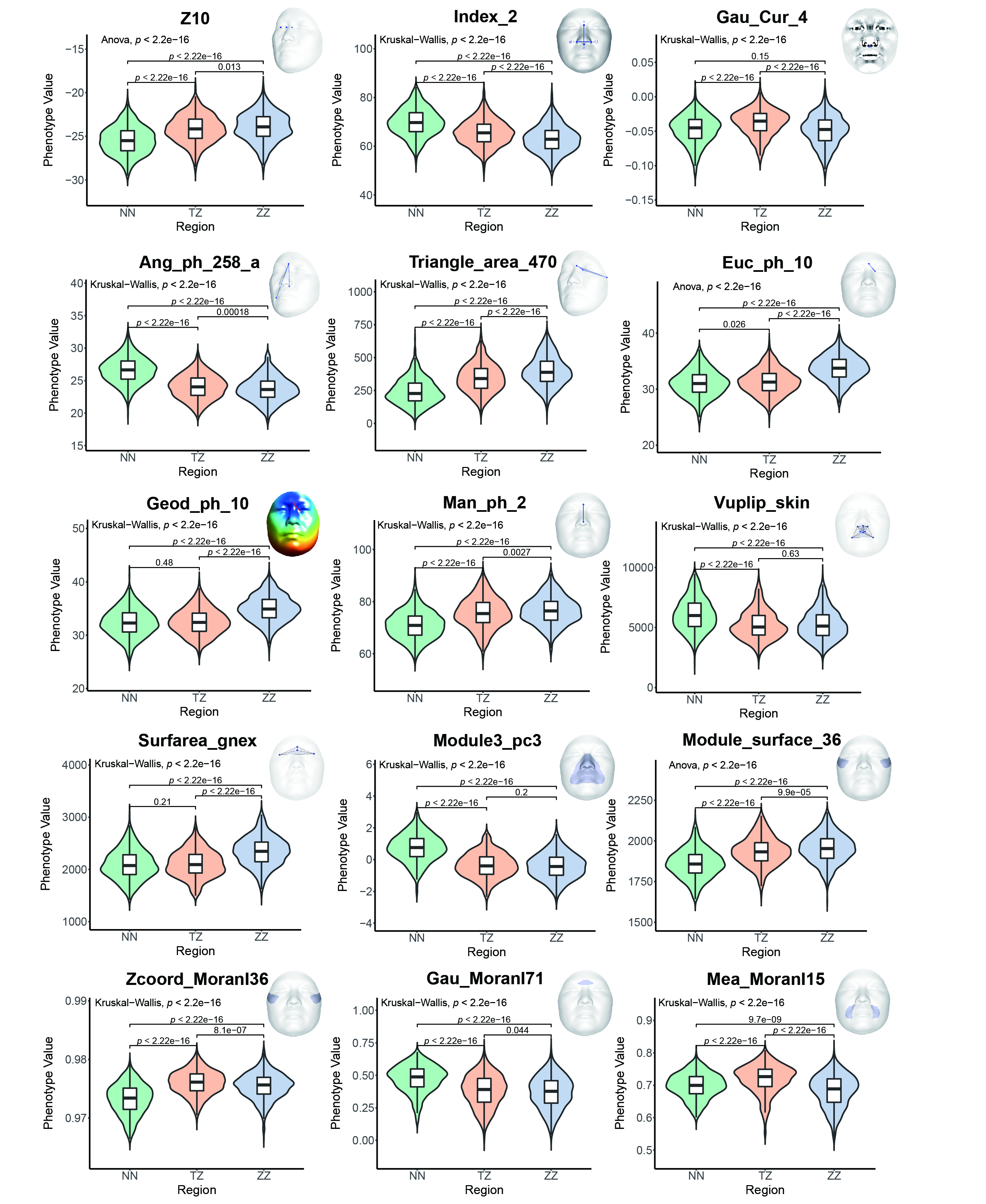
**

**Supplementary Fig. 4** Violin plots of 15 heterogeneous phenotypes among Nanning (NN), Taizhou (TZ), and Zhenzhou (ZZ). The inner boxes and lines in the violin plot align with the boxes and whiskers in the box plot. In a box plot, the central line in the box indicates the median of the distribution, whereas the top and bottom of the box represent the third and first quartiles of the data, respectively. The *p* value at the top includes a test of the total difference among the three regional groups and a comparison between each two

**Homogeneity of facial features among Han Chinese**

**
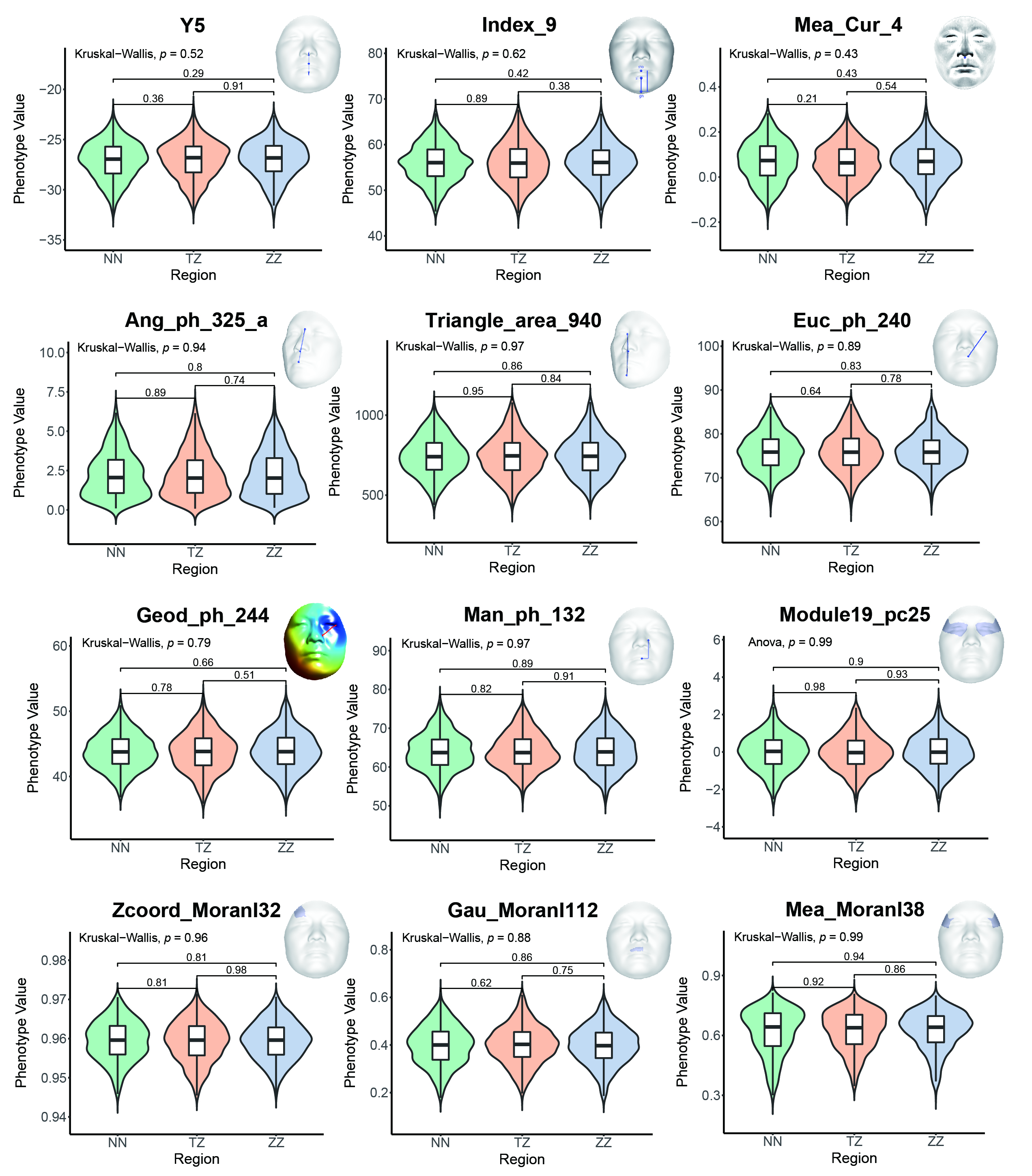
**

**Supplementary Fig. 5** Violin plots of 12 homogeneous phenotypes among Nanning (NN), Taizhou (TZ), and Zhenzhou (ZZ). The inner boxes and lines in the violin plot align with the boxes and whiskers in the box plot. In a box plot, the central line in the box indicates the median of the distribution, whereas the top and bottom of the box represent the third and first quartiles of the data, respectively. The *p* value at the top includes a test of the total difference among the three regional groups and a comparison between each two

***Supplementary Tables***

**Supplementary Table 1** Summary of facial landmarks

| Landmark | Abbreviation | Definition |
| --- | --- | --- |
| Glabella | g | The most prominent point on the frontal bone above the root of the nose. |
| Nasion | n | Midline point where the frontal and nasal bones contact. Corresponds to the underlying bony landmark. |
| Pronasale | prn | Midline point marking the maximum protrusion of the nasal tip. |
| Subnasale | sn | Midline point marking the junction between the inferior border of the nasal septum and the cutaneous upper lip. |
| Labrale Superius | ls | Midline point of the vermilion border of the upper lip, at the base of the philtrum. |
| Stomion | sto | Midpoint of the labial fissure. |
| Labrale Inferius | li | Midline point of the vermilion border of the lower lip. |
| Sublabiale | sl | Midpoint along the inferior margin of the cutaneous lower lip. |
| Gnathion | gn | Midline point on the inferior border of the mandible. Corresponds to the underlying bony landmark. |
| Endocanthion (right) | en_r | Apex of the angle formed at the inner corner of the palpebral fissure where the upper and lower eyelids meet. |
| Endocanthion (left) | en_l | Same as endocanthion (right). |
| Exocanthion (right) | ex_r | Apex of the angle formed at the outer corner of the palpebral fissure where the upper and lower eyelids meet. |
| Exocanthion (left) | ex_l | Same as exocanthion (right). |
| Alare (right) | al_r | Most lateral point on the nasal ala. |
| Alare (left) | al_l | Same as alare (right). |
| Subalare (right) | sbal_r | Point located at the lower margin of the nasal ala, where the cartilage inserts in the cutaneous upper lip. |
| Subalare (left) | sbal_l | Same as subalare (right). |
| Crista Philtri (right) | cph_r | Point marking the lateral crest of the philtrum at the vermilion border of the upper lip. |
| Crista Philtri (left) | cph_l | Same as crista philtri (right). |
| Cheilion (right) | ch_r | Point marking the lateral extent of the labial fissure. |
| Cheilion (left) | ch_l | Same as cheilion (right). |
| Superior Alar Groove (right) | supa_r | Most superior portion of alar groove. |
| Superior Alar Groove (left) | supa_l | Same as superior alar groove (right). |
| Zygion (right) | zy_r | Most prominent portion of zygomatic arch |
| Zygion (left) | zy_l | Same as zygion (right). |
| Pogonion | p | Most prominent portion of chin, anatomical pogonion. |

**Supplementary Table 2** Mean x, y, and z differences between twice manual landmarking on 3D face (mm)

| **Landmark (Abbr)** | **Mean x difference (SD)** | **Mean y difference (SD)** | **Mean z difference (SD)** |
| --- | --- | --- | --- |
| Glabella (g) | 0.64 (0.83) | 2.26 (1.85) | 0.18 (0.21) |
| Nasion (n) | 0.65 (0.83) | 2.07 (1.74) | 0.38 (0.41) |
| Pronasale (prn) | 0.66 (0.82) | 0.77 (0.88) | 0.11 (0.16) |
| Subnasale (sn) | 0.68 (0.83) | 0.59 (0.61) | 0.64 (0.77) |
| Labrale superius (ls) | 0.65 (0.87) | 0.66 (0.84) | 0.17 (0.23) |
| Stomion (sto) | 0.66 (0.87) | 0.51 (0.64) | 0.38 (0.54) |
| Labrale inferius (li) | 0.65 (0.88) | 0.66 (0.87) | 0.11 (0.18) |
| Sublabiale (sl) | 0.66 (0.87) | 1.02 (1.06) | 0.36 (0.48) |
| Gnathion (gn) | 0.67 (0.85) | 1.05 (1.06) | 0.91 (0.98) |
| Right endocanthion (en_r) | 1.38 (1.26) | 0.74 (0.73) | 0.56 (0.59) |
| Left endocanthion (en_l) | 1.02 (1.01) | 0.77 (0.78) | 0.50 (0.54) |
| Right exocanthion (ex_r) | 1.76 (1.35) | 0.75 (0.63) | 1.56 (1.42) |
| Left exocanthion (ex_l) | 1.49 (1.23) | 0.96 (0.77) | 1.60 (1.40) |
| Right alare (al_r) | 0.46 (0.43) | 0.96 (0.99) | 2.02 (2.19) |
| Left alare (al_l) | 0.47 (0.44) | 1.10 (1.06) | 1.41 (1.83) |
| Right subalare (sbal_r) | 0.72 (0.85) | 0.49 (0.66) | 0.25 (0.38) |
| Left subalare (sbal_l) | 0.72 (0.85) | 0.58 (0.69) | 0.29 (0.38) |
| Right crista philtri (cph_r) | 1.47 (1.27) | 0.76 (0.87) | 0.37 (0.35) |
| Left crista philtri (cph_l) | 1.00 (0.96) | 0.75 (0.87) | 0.28 (0.28) |
| Right cheilion (ch_r) | 1.43 (1.18) | 0.61 (0.57) | 1.13 (1.09) |
| Left cheilion (ch_l) | 1.36 (1.17) | 0.70 (0.64) | 0.92 (0.89) |
| Right superior alar groove (supa_r) | 0.95 (0.79) | 0.94 (0.86) | 1.57 (1.37) |
| Left superior alar groove (supa_l) | 0.83 (0.77) | 0.99 (0.95) | 1.04 (0.99) |
| Right zygion (z_r) | 0.84 (0.71) | 3.91 (3.50) | 4.68 (4.07) |
| Left zygion (z_l) | 0.89 (0.81) | 3.89 (3.49) | 4.83 (4.09) |
| Pogonion (p) | 0.68 (0.87) | 1.60 (1.57) | 0.19 (0.32) |

High reproducibility (SD < 0.5 mm); Moderate reproducibility (0.5 ≤ SD ≤ 1 mm); Poor reproducibility (SD > 1 mm)

**Supplementary Table 3** Intraclass correlation coefficient (ICC) between spot sampling and 3D manual landmarking

| **Phenotype** | [**Spot sampling**](http://www.baidu.com/link?url=PkPRwxWy-w4ngvt4E1iJbJs9E3IBFaIqyGVbPeNbOy0biMX3VXY0R5cKNSa0DmoyMK1LdOmmGONRYW6XgRktDh3LUrMvGFDrR3HAS9eqrhFnzbC_v8YAV-MYY-LEK549)  **(M ± SD)** | **Manual**  **(M ± SD)** | **ICC** | **ICC**  **lower** | **ICC**  **upper** | **F** | **df1** | **df2** | ***p* value** |
| --- | --- | --- | --- | --- | --- | --- | --- | --- | --- |
| Face breadth | 140.98 ± 7.09 | 145.16 ± 7.56 | 0.91 | 0.90 | 0.92 | 11.28 | 2326 | 2326 | 0.000 |
| Interocular breadth | 34.12 ± 3.41 | 40.30 ± 3.14 | 0.75 | 0.73 | 0.77 | 4.02 | 2326 | 2326 | < 0.001 |
| Biocular breadth | 88.60 ± 4.73 | 85.87 ± 4.67 | 0.82 | 0.80 | 0.83 | 5.50 | 2326 | 2326 | 0.000 |
| Morphological facial height | 117.67 ± 7.56 | 121.80 ± 7.36 | 0.87 | 0.86 | 0.88 | 7.62 | 2326 | 2326 | 0.000 |
| Nasal height | 52.59 ± 4.36 | 59.60 ± 4.14 | 0.78 | 0.76 | 0.80 | 4.59 | 2326 | 2326 | < 0.001 |
| Nose length | 45.12 ± 4.44 | 50.72 ± 4.16 | 0.87 | 0.86 | 0.88 | 7.65 | 2326 | 2326 | 0.000 |
| Alare height | 13.42 ± 2.01 | 15.69 ± 1.41 | 0.64 | 0.61 | 0.67 | 2.81 | 2326 | 2326 | < 0.001 |
| Nose breadth | 37.18 ± 3.28 | 39.31 ± 3.40 | 0.90 | 0.89 | 0.91 | 9.72 | 2326 | 2326 | 0.000 |
| Nasal depth | 12.79 ± 3.30 | 11.51 ± 1.99 | 0.60 | 0.56 | 0.63 | 2.47 | 2326 | 2326 | < 0.001 |
| Subnasale gnathion distance | 64.12 ± 5.80 | 63.68 ± 5.14 | 0.87 | 0.86 | 0.88 | 7.58 | 2326 | 2326 | 0.000 |
| Upper lip skin height | 16.66 ± 2.97 | 17.35 ± 2.49 | 0.84 | 0.82 | 0.85 | 6.17 | 2326 | 2326 | 0.000 |
| Lip height | 15.88 ± 3.42 | 14.59 ± 1.81 | 0.74 | 0.72 | 0.76 | 3.83 | 2326 | 2326 | < 0.001 |
| Thickness of lips | 7.43 ± 1.65 | 8.30 ± 1.33 | 0.69 | 0.66 | 0.71 | 3.21 | 2326 | 2326 | < 0.001 |
| Mouth breadth | 47.78 ± 4.07 | 48.59 ± 3.63 | 0.79 | 0.78 | 0.81 | 4.83 | 2326 | 2326 | < 0.001 |
| Morphological upper facial height | 69.24 ± 5.82 | 76.76 ± 5.25 | 0.82 | 0.80 | 0.83 | 5.53 | 2326 | 2326 | 0.000 |
| Height of the upper face | 76.67 ± 5.99 | 83.64 ± 5.41 | 0.81 | 0.79 | 0.83 | 5.27 | 2326 | 2326 | < 0.001 |
| Height of the upper lip | 24.09 ± 3.19 | 24.29 ± 2.69 | 0.83 | 0.81 | 0.84 | 5.85 | 2326 | 2326 | 0.000 |
| Vermilion height of the lower lip | 8.45 ± 2.35 | 6.93 ± 1.12 | 0.68 | 0.65 | 0.70 | 3.11 | 2326 | 2326 | < 0.001 |
| Height of the mandible | 40.04 ± 5.37 | 40.23 ± 3.89 | 0.72 | 0.70 | 0.74 | 3.57 | 2326 | 2326 | < 0.001 |

Consistency standard, ICC < 0.5 (poor); 0.5 ≤ ICC < 0.75 (moderate); 0.75 ≤ ICC ≤ 0.9 (good); ICC > 0.9 (excellent), where *p* value 0.000 represents 5.0E-324 over the minimum integrity

**Supplementary Table 4** Adonis tests of significant differences among Taizhou, Zhengzhou, and Nanning

| Group | Adonis | | |
| --- | --- | --- | --- |
|  | **df** | **R^2^** | ***p* value** |
| TZ/ZZ | 1 | 0.007 | 0.003* |
| TZ/NN | 1 | 0.042 | 0.003* |
| ZZ/NN | 1 | 0.054 | 0.003* |

The Adonis test is a nonparametric MANOVA. R^2^ represents the degree of variation between groups that was explained. The credibility of this test is high (* represents Bonferroni multiple correction *p* value, *p* < 0.017). Cross-validation of Adonis models obtained from 999 permutation tests. The R value was calculated based on the Euclidean dissimilarity (*p* < 0.05). The closer the R value was to 1, the larger the difference between the groups compared with the difference within the groups (*p* < 0.05). TZ, Taizhou; ZZ, Zhengzhou; NN, Nanning

**Supplementary Table 5** Adonis tests of significant differences among Taizhou, Zhengzhou, and Nanning male Han

| Group | Adonis | | |
| --- | --- | --- | --- |
|  | **df** | **R^2^** | ***p* value** |
| Total | 2 | 0.07 | 0.001 |
| TZ/ZZ | 1 | 0.01 | 0.006* |
| TZ/NN | 1 | 0.065 | 0.003* |
| ZZ/NN | 1 | 0.072 | 0.003* |

The Adonis test is a nonparametric MANOVA. R^2^ represents the degree of variation between groups that was explained. The credibility of this test is high (*p* < 0.05; * represents Bonferroni multiple correction *p* value, *p* < 0.017). Cross-validation of Adonis models obtained from 999 permutation tests. The R value was calculated based on the Euclidean dissimilarity (*p* < 0.05). The closer the R value was to 1, the larger the difference between the groups compared with the difference within the groups (*p* < 0.05). TZ, Taizhou; ZZ, Zhengzhou; NN, Nanning

**Supplementary Table 6** Adonis tests of significant differences among Taizhou, Zhengzhou, and Nanning female Han

| Group | Adonis | | |
| --- | --- | --- | --- |
|  | **df** | **R^2^** | ***p* value** |
| Total | 2 | 0.08 | 0.001 |
| TZ/ZZ | 1 | 0.011 | 0.003* |
| TZ/NN | 1 | 0.078 | 0.003* |
| ZZ/NN | 1 | 0.094 | 0.003* |

The Adonis test is a nonparametric MANOVA. R^2^ represents the degree of variation between groups that was explained. The credibility of this test is high (*p* < 0.05; * represents Bonferroni multiple correction *p* value, *p* < 0.017). Cross-validation of Adonis models obtained from 999 permutation tests. The R value was calculated based on the Euclidean dissimilarity (*p* < 0.05). The closer the R value was to 1, the larger the difference between the groups compared with the difference within the groups (*p* < 0.05). TZ, Taizhou; ZZ, Zhengzhou; NN, Nanning

**Supplementary Table 7** Adonis tests of significant differences among Taizhou, Zhengzhou, and Nanning after adjusting for age

| Group | Adonis | | |
| --- | --- | --- | --- |
|  | **df** | **R^2^** | ***p* value** |
| Total | 2 | 0.03 | 0.001 |
| TZ/ZZ | 1 | 0.006 | 0.006* |
| TZ/NN | 1 | 0.037 | 0.003* |
| ZZ/NN | 1 | 0.033 | 0.003* |

The Adonis test is a nonparametric MANOVA. R^2^ represents the degree of variation between groups that was explained. The credibility of this test is high (*p* < 0.05; * represents Bonferroni multiple correction *p* value, *p* < 0.017). Cross-validation of Adonis models obtained from 999 permutation tests. The R value was calculated based on the Euclidean dissimilarity (*p* < 0.05). The closer the R value was to 1, the larger the difference between the groups compared with the difference within the groups (*p* < 0.05). TZ, Taizhou; ZZ, Zhengzhou; NN, Nanning
